# Combination of ESI and MALDI mass spectrometry for qualitative, semi-quantitative and *in situ* analysis of gangliosides in brain

**DOI:** 10.1038/srep25289

**Published:** 2016-05-04

**Authors:** Yangyang Zhang, Jun Wang, Jian’an Liu, Juanjuan Han, Shaoxiang Xiong, Weidong Yong, Zhenwen Zhao

**Affiliations:** 1 Beijing National Laboratory for Molecular Sciences, Key Laboratory of Analytical Chemistry for Living Biosystems, Institute of Chemistry Chinese Academy of Sciences, Beijing Mass Spectrum Center, Beijing, China; 2 Institute of Laboratory Animal Science, Chinese Academy of Medical Sciences & Peking Union Medical College, Beijing, China; 3 Graduate School, University of Chinese Academy of Sciences, Beijing, China

## Abstract

Gangliosides are a family of complex lipids that are abundant in the brain. There is no doubt the investigations about the distribution of gangliosides in brian and the relationship between gangliosides and Alzheimer’s disease is profound. However, these investigations are full of challenges due to the structural complexity of gangliosides. In this work, the method for efficient extraction and enrichment of gangliosides from brain was established. Moreover, the distribution of gangliosides in brain was obtained by matrix-assisted laser desorption ionization (MALDI) mass spectrometry imaging (MSI). It was found that 3-aminoquinoline (3-AQ) as matrix was well-suited for MALDI MS analysis of gangliosides in negative ion mode. In addition, the pretreatment by ethanol (EtOH) cleaning brain section and the addition of ammonium formate greatly improved the MS signal of gangliosides in the brain section when MALDI MSI analysis was employed. The distribution of ganliosides in cerebral cortex, hippocampus and cerebellum was respectively acquired by electrospray ionization (ESI) MS and MALDI MSI, and the data were compared for reliability evaluation of MALDI MSI. Further, applying MALDI MSI technology, the distribution of gangliosides in amyloid precursor protein transgenic mouse brain was obtained, which may provide a new insight for bioresearch of Alzheimer’s disease (AD).

In April 2013, President Obama unveiled the “BRAIN” Initiative, which was expected to uncover the mysteries of brain disorders, such as Alzheimer’s and Parkinson’s diseases, depression, post-traumatic stress disorder, and traumatic brain injury. Therefore, the development and application of innovative technologies that can elucidate the composition and change of brain, there is no doubt, will be profound. Magnetic resonance imaging (MRI) is one of the most significant contributions to the study of the structure and function of the living human brain, with a spatial resolution in the range of 1 mm for structural imaging. Mass spectrometry (MS), although cannot be directly used in the study of living body, play a crucial role in the measurement of molecular weight, identification of structure, and analysis of component change, and will hence provide great help for brain research.

Gangliosides are a large family of complex lipids that are characteristic components of neural cell membranes and abundant in the brain^1^. Gangliosides are known to provide a neuroprotective role in neuronal injury model^2^, promote neural development in the neonate^3^, and involved in memory and learning^4^,^5^,^6^,^7^. Gangliosides are also related to cancer^8^ and Alzheimer’s disease (AD)^7^. In human gliosarcoma versus normal brain tissue, the total ganglioside contents are 7.4-times lower, and exhibit a highly distinctive pattern^8^. In AD, a complex of GM1 (one subclass of ganglioside) and amyloid β-protein (Aβ) termed “GAβ,” has been found to accumulate in brain^5^. These studies show that it is of great significance to develop reliable bioanalytical methods to measure ganglioside in the brain. However, the structure of a ganglioside is very complicated, which includes a ceramide tail (fatty acid N-linked to a sphingosine) linked to a polar carbohydrate head that contains sialic acid. Among them, the numbers of carbon atoms, double bonds, and hydroxylations in the ceramide, and the numbers of saccharide residues and sialic acid are varied. The structural complexity of these lipids is an impediment to achieving accurate and precise analysis in biosystem.

So far, mass spectrometry (MS) has been extensively employed to study gangliosides. Fast atom bombardment (FAB)-MS successfully determined the structures of both the lipids and the carbohydrate portions of gangliosides standards^9^,^10^,^11^. However, FAB is a relatively harsh and insensitive ionization technique, which can cause extensive precursor ion fragmentation that complicates mixture analysis. More gentle ionization modes such as matrix-assisted laser desorption ionization (MALDI) and electrospray ionization (ESI) have also been utilized in the analysis of gangliosides. Ivleva and coworkers established a vibrationally cooled MALDI ion source, and fragile gangliosides could be desorbed from thin-layer chromatography (TLC) plates without fragmentation^12^. Further, Taki established a method for transferring lipids separated on a TLC-plate to a poly-vinylidene difluoride (PVDF) membrane and direct mass spectrometric analysis of ganglioside molecular species from the human brain by MALDI MS^13^. Recent advances in MALDI MS techniques of lipid analysis have led to the direct analysis of tissue slices with the MALDI mass spectrometry imaging (MSI). Colsch *et al.* developed a new matrix preparation (2,6-dihydroxyacetophenone [DHA]/ammonium sulfate/heptafluorobutyric acid [HFBA]) to maximize the detection of all ganglioside species, and reported the differential distribution of ganglioside species in the rat brain’s cerebrum by MALDI MSI, which was used to map and image gangliosides with detailed structural information and histological accuracy^14^,^15^. ESI is the major ionization method in MS for lipid analysis. For example, an effective method by liquid chromatography (LC) ESI tandem mass spectrometry (LC/ESI-MS/MS) were developed to separate and distinguish regioisomeric gangliosides, such as GM1a and -1b, GD1a, -1b, and -1c, and GT1a, -1b, and -1c^16^.

Although MS is a powerful analytical tool, each ionization method still has some limits. MALDI MS analysis has the disadvantages of rather poor reproducibility, mainly originating from the heterogeneity of the matrix-analyte crystals, which leads to MALDI MS being heavily criticized for its quantitative analysis^17^. In addition, the samples will be quickly consumed under laser irradiation on specific site, which lead to a discontinuous ion flow, and is adverse to qualitative analysis. Meanwhile, the ESI MS cannot be directly used for imaging. The desorption electrospray ionization (DESI) may be capable of *in situ* analysis^18^, however the convenience and spatial resolution for imaging is expected to be improved. In our work, we constructed a workflow, by combination ESI with MALDI mass spectrometry, for qualitative, semi-quantitative and *in situ* analysis of gangliosides in brain. We performed cross validation of data for method validation. We expect to maximize the advantages of each ionization method.

## Results

### The Work Flow for Qualitative, Semi-quantitative and in Situ Analysis of Gangliosides

**Figure 1:**
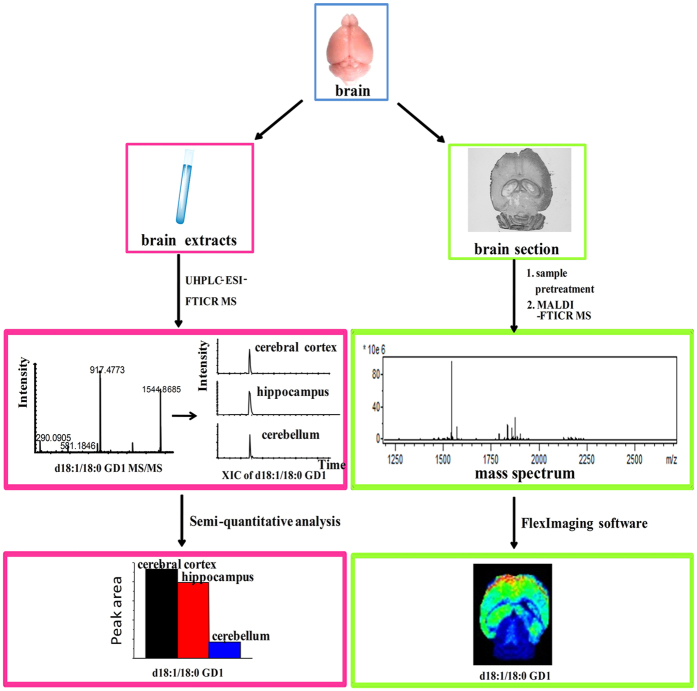
The workflow for analysis of gangliosides by combining ESI with MALDI MS.

Mass spectrometry (MS), in particular MALDI and ESI MS, has been extensively employed to study lipids^19^,^20^,^21^. MALDI MS has demonstrated its unique features, namely, no necessity of labeling, high sensitivity, high throughout, molecule-specific, and the capacity of *in situ* localizing a wide range of biomolecules simultaneously from a tissue specimen in one single run. However, MALDI MS analysis has the disadvantages of both qualitative and quantitative analysis, already mentioned in Introduction. In this regard, ESI MS could be a perfect complementary. In this work, we constructed a workflow, shown in Fig. 1, by combination ESI with MALDI MS, for qualitative, semi-quantitative and *in situ* analysis of gangliosides in brain. The ESI and MALDI MS complement and verify each other, and we expect to maximize the advantages of each ionization method and to obtain more comprehensive and more accurate data as far as possible.

### ESI MS Analysis of Gangliosides

Using ganglioside standards, the efficiency of extraction was investigated. The efficiency of extraction was calculated by comparison the peak area after extraction with the peak area without extraction. The efficiency of all gangliosides (GM1, GM3, GD1 and GT1) is from 35.5% to 89.2%. The efficiency of extraction of d18:1/16:0, d18:1/18:0, d18:1/20:0, d18:1/21:0, d18:1/22:0, and d18:1/24:0 GM3 were 76.8%, 68.0%, 67.2%, 57.4%, 55.8% and 35.5%, respectively. In addition, the efficiency of extraction of d18:1/18:0 and d18:1/20:0 GM1 were 85.4% and 86.8%, respectively, meanwhile, the efficiency of extraction of d18:1/18:0 and d18:1/20:0 GD1 were 86.2% and 86.5%, the efficiency of extraction of d18:1/18:0 and d18:1/20:0 GT1 were 88.2% and 87.3%, respectively. These data suggested that the efficiency of extraction was depended on the length of fatty acid chain in ceramide portion and the number of saccharide residues and sialic acid. Basically, the shorter the fatty acid chain in ceramide portion and the more the number of saccharide residues and sialic acid, the greater the polarity of ganglioside and the higher the efficiency of extraction. In addition, the detection limit of ganglioside is around 1 μM in negative-ion detection mode.

**Figure 2:**
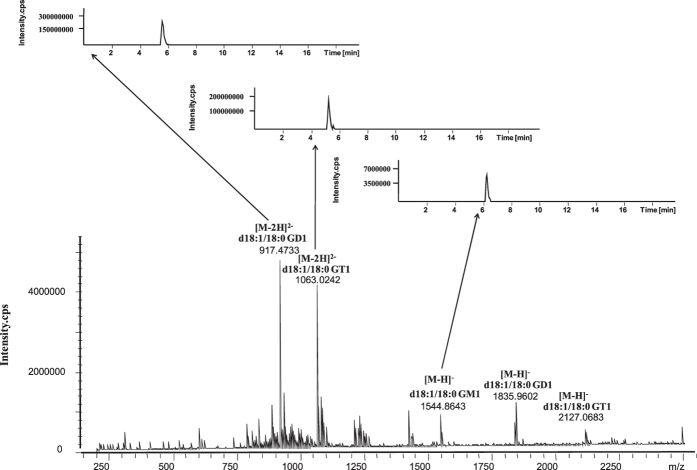
The mass spectrum and its corresponding extracted ion chromatogram (XIC) obtained by UHPLC-ESI-FTICR MS. The structure of a ganglioside includes a ceramide tail (fatty acid N-linked to a sphingosine) of varying length, saturation and hydroxylation linked to a polar carbohydrate head that contains sialic acid. The gangliosides are grouped according to the number of their sialic acid residues: one (M), two (D), three (T) or four (Q).GM, GD and GT are main gangliosides in brain.

To overcome the phenomenon of ion suppression, UHPLC was employed to separate lipids in brain extracts before ESI MS detection. The mass spectrum and several representative extracted ion chromatogram (XIC) were shown in Fig. 2. GD, GT and GQ series mainly appeared as the doubly charged molecular ions ([M-2H]^2−^); while GM series mainly appeared as deprotonated ions ([M-H]^−^). By matching the precursor ion (mass error <5 ppm) and product ions (Supplementary Fig. S1–S8) obtained by ESI MS/MS with free online databases and standard gangliosides, gangliosides were identified, and the *m/z* value in the mass spectrum with corresponding deprotonated molecule ion and the mass accuracy after comparing the theoretical *m/z* value were indicated in Supplementary Table S1. The presence of the *O*-acetyl forms of the GD1 and GT1 gangliosides was consistent with the results reported in previous research^22^. The result showed that the major ceramide portions in brain were d18:1/18:0 and d18:1/20:0.

**Figure 3:**
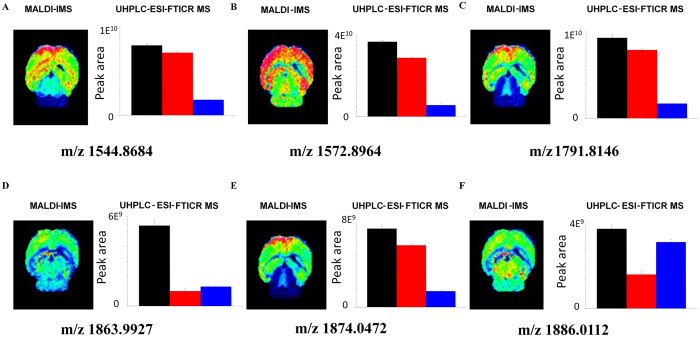
*In situ* MALDI MSI and UHPLC-ESI-FTICR MS analysis of gangliosides in brain tissues. The distribution of gangliosides with *m/z* at (**A**) 1544.8684 (**B**) 1572.8964 (**C**) 1791.8146 (**D**) 1863.9927 (**E**) 1874.0472 and (**F**) 1886.0112 in cerebral cortex (black histogram), hippocampus (red histogram) and cerebellum (blue histogram) from mouse brain obtained by MALDI MSI were confirmed by UHPLC-ESI-FTICR MS results.

The peak area of extracted ion in XIC could be used for semi-quantitative analysis of the ganglioside. The opposing polarities of head group and ceramide tail of gangliosides required finely balanced proportions of organic solvents to concomitantly meet the requirements for efficient solubilization, chromatographic separation, and ionization. The optimized LC conditions were shown in “FTICR MS” in Methods below. Due to the high mass accuracy and resolution, we could extract an exact mass ion chromatogram with a narrow mass window of 5 ppm. The retention time (RT) of gangliosides identified was also listed in Supplementary Table S1. The data indicate that the ganglioside was separated on the basis of the ceramide portion, and the gangliosides with long and less-unsaturated fatty acid chain in ceramide portion eluted later. The relative standard deviation (RSD) of peak area of every single ganglioside is less than 7%. The peak areas in XIC of each ganglioside in cerebral cortex, hippocampus and cerebellum were compared, and a different distribution of ganglioside was observed. For example, d18:1/18:0 GM1 (*m/z* 1544.8643) and d18:1/20:0 GM1 (*m/z* 1572.8964), is mainly present in cerebral cortex and hippocampus, less expressed in cerebellum; however, d18:1/20:0 GD1 (*m/z* 1863.9927) is mostly found in cerebral cortex, barely present in hippocampus and cerebellum. For these gangliosides together with GalNAcβ1-4(NeuGcα2-3)Galβ1-3GalNAcβ1-4Galβ1-4Glcβ-Cer(d18:1/20:0) (*m/z* 1791.8146) and NeuAcα2-8NeuAcα2-8NeuAcα2-3Galβ1-4Glcβ-Cer(d18:1/26:0) (*m/z* 1874.0472) and 9-OAc-NeuAcα2-8NeuAcα2-8NeuAcα2-3Galβ1-4Glcβ-Cer(d18:1/24:1(15Z)) (*m/z* 1886.0112), the detailed distribution information in cerebral cortex, hippocampus and cerebellum from C57BL/6 control mice, as representatives, was showed in Fig. 3.

### MALDI MS Analysis of Gangliosides

**Figure 4:**
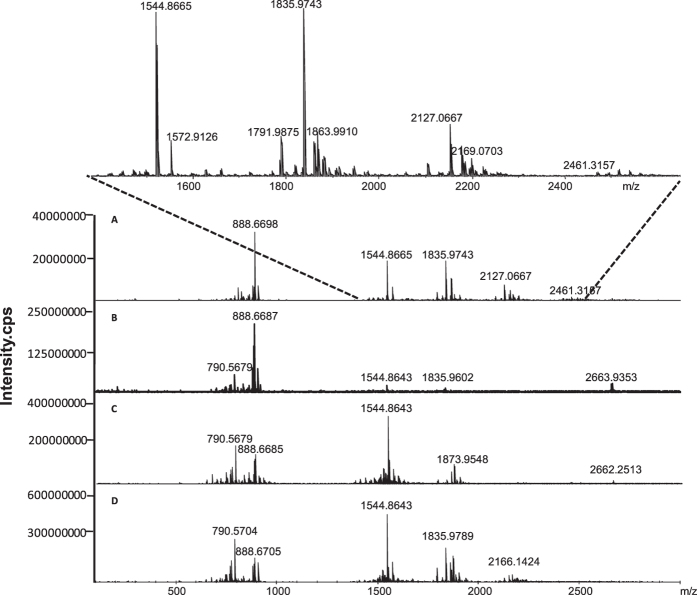
Mass spectra of mouse brain extracts analyzed in negative ion mode of MALDI-FTICR MS by using (**A**) 3-AQ, (**B**) 9-AA, (**C**) DHB and (**D**) nH as matrixes, respectively.

The matrix is a critical factor for MALDI MS analysis, and therefore we first optimized the matrix for ganglioside analysis. Among the vast majority of commercially available matrices, DHB was a widely accepted matrix compound for the analysis of lipids since it provides an excellent signal-to-noise (S/N) ratio for the peaks of the analyte of interest and provides a weak background^23^,^24^. 3-AQ, first applied by Metzger *et al.* for the analysis of plant inulins, is described as superior to DHB in terms of sensitivity and resolution in negative ion mode^25^,^26^. 9-AA is recently developed for negative charged lipids detection^27^. nH has also been reported for negative-ion MALDI of neutral oligosaccharides^28^,^29^. Therefore, these matrices were chosen to screen for the best matrix for gangliosides analysis, and the mass spectra obtained by MALDI-FTICR MS analysis of mouse brain extracts were shown in Fig. 4. Not like ESI MS, the gangliosides purely appeared as deprotonated ions ([M-H]^−^) by MALDI MS analysis. It was also observed that using 3-AQ as matrix, the mass spectrum with more gangliosides MS signals and more excellent S/N ratio was acquired. Moreover, some gangliosides MS signals, for example, the signals with *m/z* at 1791.8146, 1820.0051, 1843.9986, etc, which cannot be detected by UHPLC-ESI-FTICR MS were observed. By matching the precise precursor ion obtained by FTICR MS with free online databases and MALDI MS/MS spectra (Supplementary Fig. S9), gangliosides were identified and listed in Table 1. These promising features allow the matrix 3-AQ to be well-suited for MALDI MS analysis of gangliosides in negative mode, and therefore the 3-AQ was used as the matrix for MALDI MSI analysis of ganglioside in brain section.

**Figure 5:**
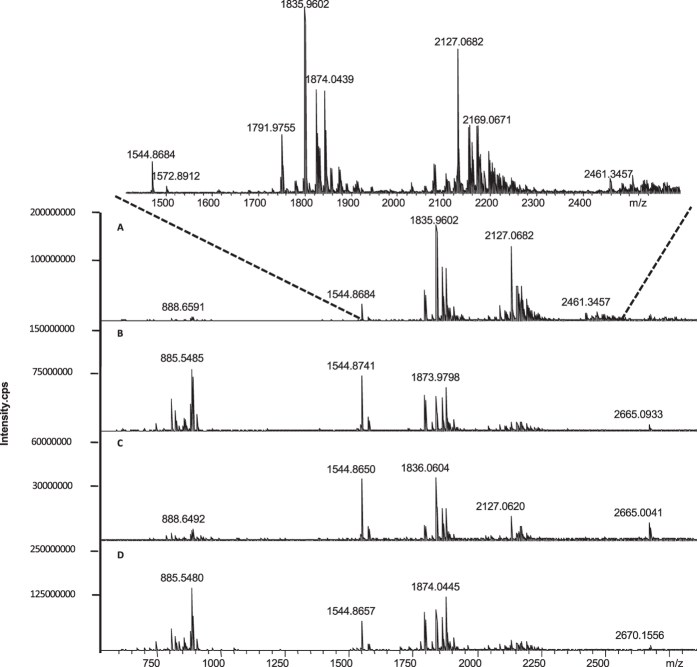
Mass spectra of brain tissue sections obtained by MALDI-FTICR MS in negative ion mode. (**A**) Brain tissue section was pretreated by 70% EtOH and 95% EtOH each for 30 s and 3-AQ (30 mg/mL)/ammonium formate (125 mM) as matrix; (**B**) No treatment for brain tissue section, and 3-AQ used as matrix; (**C**) Brain tissue section was pretreated similarly with A, but no ammonium formate was used; (**D**) No treatment for brain tissue section, and 3-AQ (30 mg/mL)/ammonium formate (125 mM) as matrix.

The sample preparation is important for MALDI MSI analysis. We first tried to use the 3-AQ as matrix for MALDI MSI analysis of ganglioside in brain section (Fig. 5B). Unfortunately, there was nearly no gangliosides signal but full of phosphatidylserines (PSs) and phosphatidylinositols (PIs). One work reported that the phospholipid, like phosphatidylcholine, would suppress other lipid’s detection, and a NH_4_Ac wash removes contaminants in tissue, enhances the overall spectral quality, and benefits additionally in profiling of biological molecules in tissue^30^. Therefore, different solvents, including chloroform, methanol and ethanol were used to clean the tissue section. It was found that a pretreatment of cleaning by 70% EtOH for 30 s and 95% EtOH again for 30 s would greatly improve the ganglioside signal (Fig. 5C). More recently, it has been reported that addition of ammonium sulfate to the 2,6-dihydroxyacetophenone (DHA) matrix in sample preparation minimized salt adducts and increased detection of deprotonated ions [M-H]^−^ for all ganglioside species including minor and *O*-acetylated species^14^; therefore some ammonium salts were tested as additive of matrix, and we found that adding ammonium formate to matrix can make the signal at *m/z* >2000 strengthened, including d18:1/18:0 GT1(*m/z* 2127.0682), d18:1/20:0 GT1(*m/z* 2155.0903), *O*-Acetyl d18:1/18:0 GT1(*m/z* 2169.0694) and *O*-Acetyl d18:1/20:0 GT1(*m/z* 2197.1045) (Fig. 5D). Based on the above, we had brain tissue section pretreated by 70% EtOH, 95% EtOH each for 30 s and 3-AQ/125 mM ammonium formate as matrix, which gave rise to more and higher gangliosides MS signals, like d18:1/18:0 GQ1(*m/z* 2418.1509) and d18:1/20:0 GQ1(*m/z* 2446.1805) (Fig. 5A). However, these pretreatments may cause problems. One concern is that the application of EtOH washing step would remove some gangliosides from brain section. So the EtOH after washing were collected, dried and re-suspended by 10 μL of a mixture of methanol and water (1:1, v/v) for analysis by MALDI FTICR MS in negative ion detection mode by using 3-AQ as matrix. The result showed (Supplementary Fig. S10) that no gangliosides were detected, showing that the application of EtOH washing step didn’t remove gangliosides. Another concern is that the EtOH treatment would affect the distribution of gangliosides in the brain section. Therefore, the effect of EtOH on the distribution of gangliosides was carefully checked. The results were shown in Supplementary Fig. S11. The distribution of gangliosides with or without EtOH cleanup was almost identical. After cleanup by EtOH, the MS signals of gangliosides were much higher (Fig. 5A). Taken together, EtOH cleanup was adopted.

Using MALDI MSI, the distributions of gangliosides in cerebral cortex, hippocampus and cerebellum of C57BL/6 control mice brain of about 20 gangliosides were acquired, and the mappings of gangliosides with *m/z* at 1544.8643, 1572.8964, 1791.8146, 1863.9927, 1874.0472 and 1886.0112, as representative, were shown in Fig. 3. These results were consistent with those obtained by UHPLC-ESI-FTICR MS, suggesting that the data obtained by MALDI MSI was reliable.

### MALDI MSI Analysis of Gangliosides in Brain with Alzheimer’s Disease (AD)

In this work, ESI and MALDI mass spectrometry were combined for the analysis of ganglioside composition and their corresponding change in brain. It was reported that ganglioside metabolism is closely associated with the pathology of Alzheimer’s disease (AD)^5^,^7^. Based on these studies and our research purpose, amyloid precursor protein (APP) transgenic mouse model, a well-known AD mouse model, was used.

**Figure 6:**
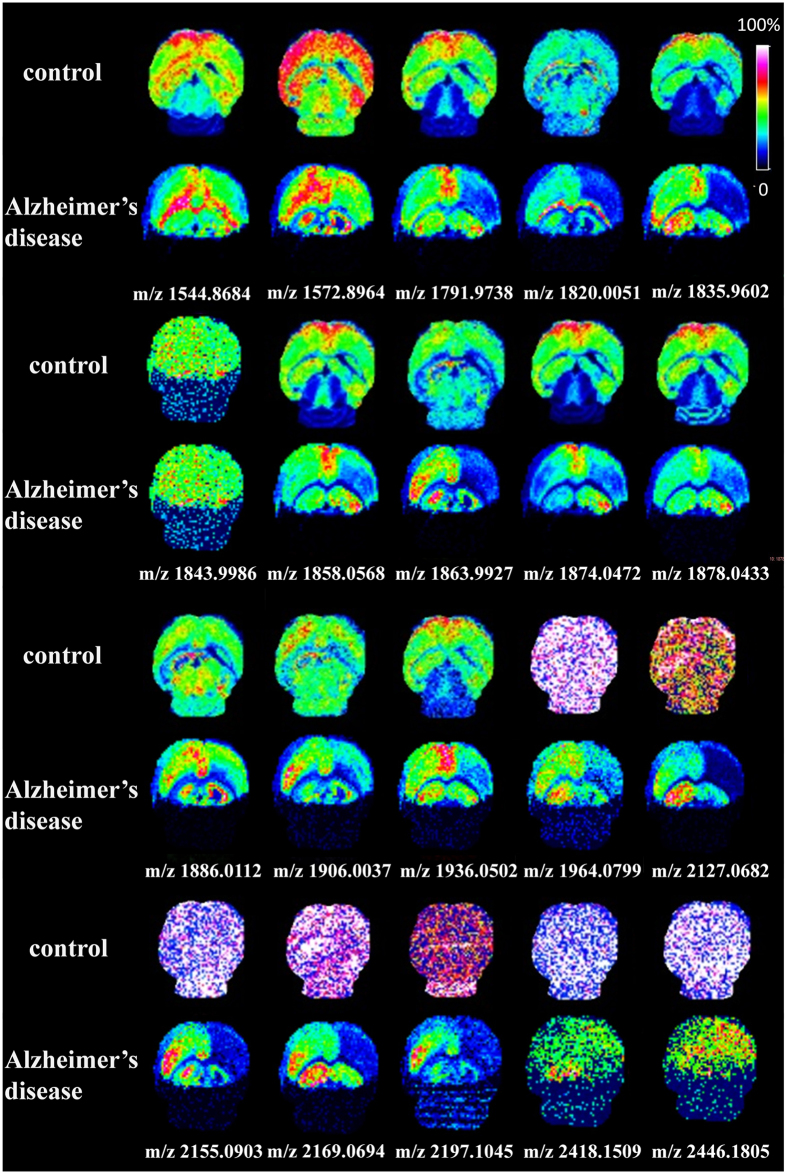
*In situ* MALDI MSI analysis of gangliosides in mouse brain from control group and Alzheimer’s disease (AD) group. Mouse brains were removed and immediately frozen under −80 °C. Sectioned brain slices at 12 μm thickness were pretreated and then used for *in situ* imaging. Mass imaging data were acquired in negative ionization mode with 200 μm spatial resolution.

APP has been known to act as an important protein during the occurrence of AD. To explore the brain ganglioside alterations in AD, 5-month old mice (3 pairs), total 6 mice, were used for MALDI MSI analysis. The MALDI MSI results were very consistent and shown in Fig. 6. In the case of AD group, we can clearly find that 20 kinds of gangliosides in cerebellum were nearly disappeared. After careful observation, we also found that lots of gangliosides with *m/z* at 1572.8964, 1791.8146, 1820.0051, 1835.9602, 1858.0568, 1863.9927, 1878.0433, 1886.0112, 1906.0037, 1936.0502, 1964.0799, 2127.0682, 2155.0903, 2169.0694 and 2197.1045 had lower distribution in right cerebral hemispheres of AD group.

**Figure 7:**
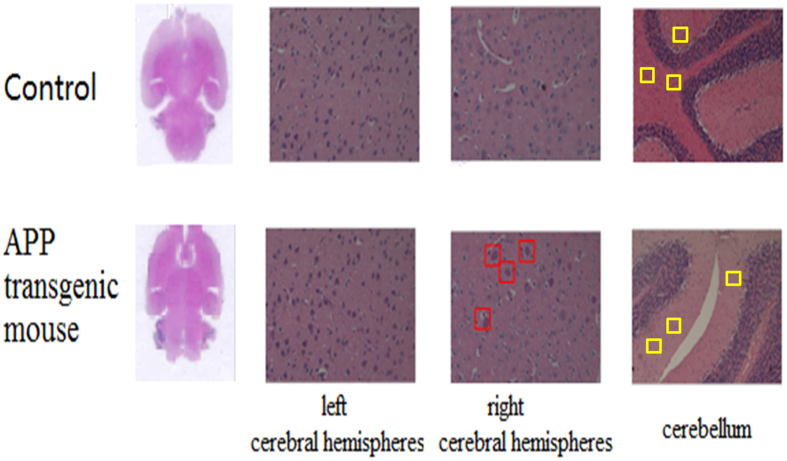
Optical images of corresponding H&E stained sections of control and APP transgenic mouse brain. The accumulations of amyloid β (Aβ) protein were indicated in red box. The neuronal cells (blue dot) were indicated in yellow box.

H&E staining experiments were performed. The histopathological features (Fig. 7) from the examination showed that amyloid β (Aβ) protein were accumulated in right cerebral hemispheres of APP transgenic mouse brain. In addition, compared with the cerebellum of control mouse, neuronal cells (blue dot) become significantly reduced in APP transgenic mouse. The damage originated from the amyloid β (Aβ) protein accumulation in right cerebral hemispheres, and the lack of neuronal cells in cerebellum, might be the reason that the gangliosides were reduced or disappeared in right cerebral hemispheres and cerebellum of APP transgenic mouse brain, respectively. The low unsaturated gangliosides content in right cerebral hemispheres of APP mice may also be a result of Aβ-mediated lipid oxidation. Unsaturated lipids in the brain are readily attacked by free radicals because of their double bond content, becoming oxidized into lipid peroxides^31^. Aβ, the primary component of amyloid plaques, has been implicated in oxidative damage through lipid peroxidation in synaptic plasma membranes^32^,^33^. Indeed, increased lipid peroxidation has been observed in human AD, mild cognitive impairment, and in transgenic mouse models of AD^34^,^35^,^36^, suggesting that antioxidant defenses are impaired in AD^37^. These changes of gangliosides in different area of the brain could contribute to the development of the AD. The present data strongly suggest that application of this technology to gangliosides analysis from pathological brain may provide new insight for bioresearch of AD.

## Discussion

A workflow by combination ESI with MALDI MS, for qualitative, semi-quantitative and *in situ* analysis of gangliosides in brain was developed. The extraction efficiency of gangliosides by Bligh and Dyer method was investigated, and the efficiency was from 35.5% to 89.2%, depending on the structure and property of the gangliosides. A FTICR MS was selected for this study due to the high mass accuracy and resolution, which greatly improved the accuracy of qualitative analysis. The sample preparation was optimized for MALDI MSI analysis, and it was found that 3-AQ (30 mg/mL)/ammonium formate (125 mM) as the matrix, and pretreatment by EtOH cleaning brain section could be used for MALDI MSI analysis of ganglioside in brain section. The result of gangliosides distribution obtained by MALDI MSI was confirmed by UHPLC-ESI MS, suggesting that the data obtained by MALDI MSI was reliable.

To explore the potential feasibility of our approach, the brain ganglioside alterations in Alzheimer’s disease mouse were investigated. The distribution of gangliosides in brain was obtained, which firstly showed that gangliosides were almost nonexistent in the cerebellum of amyloid precursor protein (APP) transgenic mouse brain, in addition, most of gangliosides had lower distribution in right cerebral hemispheres of APP group. The altered distribution of gangliosides may be related with the damage originated from the amyloid β (Aβ) protein accumulation in right cerebral hemispheres, and the lack of neuronal cells in cerebellum, which may provide a new insight for bioresearch of Alzheimer’s disease (AD).

To the best of our knowledge, this is the first report to combine ESI with MALDI MS for analysis of gangliosides. The ESI and MALDI MS complement and verify each other to obtain more comprehensive and more accurate data as far as possible. This workflow is believed to maximize the advantages of ESI with MALDI MS and to complement or verify with each other, and is expected to provide great help for brain research.

## Methods

### Chemicals and Reagents

Standard lipids, including total ganglioside extracts (porcine brain, ammonium salt, Product No.: 860053), ganglioside GM1 (ovine brain, Product No.: 860065), ganglioside GM3 (bovine milk, Product No.: 860058) and ganglioside GD3 (bovine milk, Product No.: 860060) were purchased from Avanti Polar Lipids (Birmingham, AL, USA). HPLC-grade methanol (MeOH), ethanol (EtOH), isopropanol (IPA), acetonitrile (CH_3_CN), chloroform (CHCl_3_), formic acid (FA) as well as ammonium formate (HCOONH_4_) were purchased from Sigma-Aldrich (St. Louis, MO, USA) or Fisher Scientific (Pittsburgh, PA, USA). The matrix 3-aminoquinoline (3-AQ), 9-aminoacridine (9-AA) were purchased from Lancaster (Morecambe, UK), 2,5-dihydroxybenzoic acid (DHB) was purchased from Acros (Pittsburgh, PA, USA) and norharmane (nH) were purchased from Sigma-Aldrich (St. Louis, MO, USA). Ultra-pure water was obtained from a Milli-Q purification system (Millipore Corporation, USA). All of the above materials were used as received without further purification.

### Animals

The amyloid precursor protein (APP) transgenic mice and controls (C57BL/6 mice, 5 months old, Female, 6 pairs) were provided by Dr. Weidong Yong in the institute of Laboratory Animal Science, Chinese Academy of Medical Sciences. The mice were sacrificed by suffocation of CO_2_, and their brains were immediately surgically removed. The cerebral cortex, hippocampus and cerebellum tissues were manually separated and quickly frozen in liquid nitrogen. The whole brain from APP transgenic mice and controls were as well quickly frozen in liquid nitrogen, or fixed by 10% formalin solution for hematoxylin and eosin (H&E) staining. These tissues were stored at −80 °C until use. The animal experiments were performed according to the ‘Guide for the Care and Use of Laboratory Animals’ and were approved by the Animal Care and Use Committee of the Chinese Academy of Sciences.

### Lipid Extraction

Frozen tissues, including cerebral cortex, hippocampus and cerebellum, were separately homogenized by using Tissuelyser-24 (Shanghai Jingxing Experimental Technology, Shanghai). Gangliosides were extracted by Bligh and Dyer method^22^,^38^,^39^. In brief, 100 μL of homogenate (water solution, totally 40 mg of the tissue) were added into 750 μL of mixture of chloroform-methanol (1:2, v:v), and then incubated in ice for 5 min. 250 μL of chloroform and 250 μL of H_2_O were then separately added. After vortexing for 1 min and centrifugation (9184 *g*, 5 min, room temperature), the upper phase was collected and the lower phase was re-extracted by adding 350 μL of H_2_O. The upper phase were combined and dried with N_2_. The gangliosides extracts were re-suspended in 100 μL of H_2_O for MS analysis.

### Tissue Sectioning

Frozen brain tissue from the APP transgenic mice and controls was fixed atop a drop of saline on the cutting stage. All brains were sectioned at 12 μm thickness using a Leica CM1950 cryostat (Leica Microsystems GmbH, Wetzlar, Germany) at −18 °C and thaw mounted onto indium tin oxide (ITO) coated glass slides (Type Ι 1.1 mm/100ea, HST Inc., Newark, NJ, USA.). Tissue sections were washed in 70% EtOH for 30 s, and then in 95% EtOH for 30 s. The glass slides were then placed into a vacuum desiccator and dried for approximately 1 h before matrix application. Finally, 3-AQ (30 mg/mL) was dissolved in CH_3_CN-H_2_O (8:2, v:v) containing 125 mM HCOONH_4_, and sprayed on the tissue section by homemade electrospray-based matrix deposition device. The tissue section was used for MALDI MSI analysis.

### FTICR MS

FTICR mass spectrometric analysis was performed with a Bruker SolariX mass spectrometer equipped with a 9.4 T superconducting magnet and SmartBeam™ laser optics. Mass calibrations were performed externally using ganglioside standards listed in **Chemicals and Reagents.** External ion accumulation was used in negative ion mode over a mass range of 200–3000 *m/z* with a resolution of 130000 at *m/z* 400. SolariX Control software was used for data acquisition.

For MALDI MS analysis, 160 laser shots were acquired per sample. For MALDI MSI analysis, mass spectra were acquired across the entire sample section with a SmartBeam II laser operating at 1000 Hz, a laser focus of 25 μm, 3 scans acquired from each matrix spot, and a raster width of 200 μm. The device parameters for both MALDI MS and MALDI MSI were chosen as follows: plate offset voltage, 100 V; deflector plate voltage, 180 V.

For ESI MS analysis, both the nebulizer and dry gases were nitrogen. Typical operating parameters were set as follows: capillary voltage, 3000 V; dry gas flow rate, 4.0 L/min; dry gas temperature, 200 °C; nebulizer gas flow rate, 1.0 bar; syringe flow rate, 180.0 μL/h. A ultra high performance liquid chromatography (UHPLC) instrument, Ultimate 3000 (Thermo), was used for loading samples and to separate gangliosides before ESI MS detection. The mobile phase A was isopropanol–acetonitrile–formic acid (90: 10: 0.1, v/v/v) containing 10 mM ammonium formate. The mobile phase B was acetonitrile–water–formic acid (70: 30: 0.1, v/v/v) containing 10 mM ammonium formate. A BEH C18 column (1.7 μm, 2.1 mm ID × 100 mm, Waters) was used for separation of gangliosides. The column was maintained at 65 °C. The UHPLC separations were 20 min per sample using the following scheme: (1) 0 min, 70% B; (2) 2 min, 57% B; (3) 2.1 min, 50% B; (4) 12 min, 46% B; (5) 12.1 min, 30% B; (6) 18 min, 1% B; (7) 18.1 min, 70% B; (8) 20 min, 70% B. All the changes are linear, and the flow rate was set to 400 μL min^−1^.

### Structural Identification

High-resolution MS spectra were used to distinguish different gangliosides, and the identification of gangliosides was achieved by precisely matching mass with free online databases, including Lipidmaps (http://www.lipidmaps.org) (accessed July 2015) and HMDB (http://www.hmdb.ca) (accessed July 2015)^40^,^41^,^42^. The mass error was set at 5 ppm. In addition, standard lipids were also used for assistance of identification of the gangliosides. MS/MS spectra obtained by collision induced dissociation (CID) were further used for confirmation of the structure of the gangliosides.

## Additional Information

**How to cite this article**: Zhang, Y. *et al.* Combination of ESI and MALDI mass spectrometry for qualitative, semi-quantitative and *in situ* analysis of gangliosides in brain. *Sci. Rep.* **6**, 25289; doi: 10.1038/srep25289 (2016).

## Supplementary Material

Supplementary Information

## Figures and Tables

**Table 1:** Profiles of mouse brain gangliosides using MALDI-FTICR MS.

Ganglioside	*m/z* Detected	*m/z* Exact	Mass accuracy/ppm
d18:1/18:0 GM1	1544.8684	1544.8694	−0.647
d18:1/20:0 GM1	1572.8964	1572.9007	−2.734
GalNAcβ1-4(NeuGcα2-3)Galβ1-3GalNAcβ1-4Galβ1-4Glcβ-Cer(d18:1/20:0)	1791.8146	1791.8166	−1.116
GalNAcβ1-4(NeuGcα2-3)Galβ1-3GalNAcβ1-4Galβ1-4Glcβ-Cer(d18:1/22:0)	1820.0051	1820.0063	−0.659
d18:1/18:0 GD1	1835.9602	1835.9648	−2.505
NeuAcα2-8NeuAcα2-8NeuAcα2-3Galβ1-4Glcβ-Cer(d18:1/24:1(15Z))	1843.9986	1844.0043	−3.091
GalNAcβ1-4(NeuAcα2-3)Galβ1-3GalNAcβ1-4Galβ1-4Glcβ-Cer(d18:1/26:1(17Z))	1858.0568	1858.0583	−0.807
d18:1/20:0 GD1	1863.9927	1863.9961	−1.824
NeuAcα2-8NeuAcα2-8NeuAcα2-3Galβ1-4Glcβ-Cer(d18:1/26:0)	1874.0472	1874.0532	−3.202
*O*-Acetyl d18:1/18:0 GD1	1878.0433	1878.0481	−2.556
9-OAc-NeuAcα2-8NeuAcα2-8NeuAcα2-3Galβ1-4Glcβ-Cer(d18:1/24:1(15Z))	1886.0112	1886.0168	−2.969
9-OAc-NeuAcα2-3Galβ1-3GalNAcβ1-4(NeuAcα2-3)Galβ1-4Glcβ-Cer(d18:1/20:0)	1906.0037	1906.0067	−1.574
NeuAcα2-3Galβ1-3GalNAcβ1-4(NeuGcα2-3)Galβ1-4Glcβ-Cer(d18:1/24:0)	1936.0502	1936.0536	−1.756
NeuAcα2-3Galβ1-3GalNAcβ1-4(NeuGcα2-3)Galβ1-4Glcβ-Cer(d18:1/26:0)	1964.0799	1964.0849	−2.546
d18:1/18:0 GT1	2127.0682	2127.0602	3.761
d18:1/20:0 GT1	2155.0903	2155.0915	−0.557
*O*-Acetyl 18:1/18:0 GT1	2169.0694	2169.0708	−0.645
*O*-Acetyl 18:1/20:0 GT1	2197.1045	2197.1021	1.092
d18:1/18:0 GQ1	2418.1509	2418.1557	−1.985
d18:1/20:0 GQ1	2446.1805	2446.1870	−2.657

## References

[b1] Svennerholm L. & Fredman P. A procedure for the quantitative isolation of brain gangliosides. Biochim. Biophys. Acta. 617, 97–109 (1980). 7353026 10.1016/0005-2760(80)90227-1

[b2] Hadjiconstantinou M. & Neff N. H. GM1 Ganglioside: *In vivo* and *in vitro* trophic actions on central neurotransmitter systems. J. Neurochem. 70, 1335–1345 (1998). 9523549 10.1046/j.1471-4159.1998.70041335.x

[b3] McJarrow P., Schnell N., Jumpsen J. & Clandinin T. Influence of dietary gangliosides on neonatal brain development. Nutrition Reviews 67, 451–463 (2009). 19674342 10.1111/j.1753-4887.2009.00211.x

[b4] Karlsson K. A. Animal glycosphingolipids as membrane attachment sites for bacteria. Annu. Rev. Biochem. 58, 309–350 (1989). 2673013 10.1146/annurev.bi.58.070189.001521

[b5] Matsuzaki K., Kato K. & Yanagisawa K. Aβ polymerization through interaction with membrane gangliosides. Biochim. Biophys. Acta 180, 868–877(2010). 20117237 10.1016/j.bbalip.2010.01.008

[b6] Futerman A. H. & Hannun Y. A. The complex life of simple sphingolipids. EMBO. J. 8, 777–782 (2004). 10.1038/sj.embor.7400208 PMC1299119 15289826

[b7] Ariga T., McDonald M. P. & Yu R. K. Role of ganglioside metabolism in the pathogenesis of Alzheimer’s disease. J. Lipid Res. 49, 1157–1175 (2008). 18334715 10.1194/jlr.R800007-JLR200 PMC2386904

[b8] Vukelic Z. *et al.* Human gliosarcoma-associated ganglioside composition is complex and distinctive as evidenced by high-performance mass spectrometric determination and structural characterization. Glycobiology 17, 504–515 (2007). 17293353 10.1093/glycob/cwm012

[b9] Egge H. *et al.* Analysis of gangliosides using fast atom bombardment mass spectrometry. Chem. Phys. Lipids 37, 127–141 (1985). 4017133 10.1016/0009-3084(85)90080-5

[b10] Ladisch S., Sweeley C. C., Becker H. & Gage D. Aberrant fatty acyl alpha-hydroxylation in human neuroblastoma tumor gangliosides. J. Biol. Chem. 264, 12097–12105 (1989). 2745431

[b11] Domon B. & Costello C. E. Structure elucidation of glycosphingolipids and gangliosides using high-performance tandem mass spectrometry. Biochemistry 27, 1534–1543 (1988). 3365408 10.1021/bi00405a021

[b12] Ivleva V. B. *et al.* Coupling thin-layer chromatography with vibrational cooling matrix-assisted laser desorption/ionization fourier transform mass spectrometry for the analysis of ganglioside mixtures. Anal. Chem. 76, 6484–6491 (2004). 15516145 10.1021/ac0491556

[b13] Taki T. An approach to glycobiology from glycolipidomics: ganglioside molecular scanning in the brains of patients with alzheimer’s disease by TLC-blot/matrix assisted laser desorption/ionization-time of flight MS. Biol. Pharm. Bull. 35, 1642–1647 (2012). 23037154 10.1248/bpb.b12-00400

[b14] Colsch B., Jackson S. N., Dutta S. & Woods A. S. Molecular microscopy of brain gangliosides: illustrating their distribution in hippocampal cell layers. ACS Chem. Neurosci. 2, 213–222 (2011). 21961052 10.1021/cn100096h PMC3181083

[b15] Colsch B. & Woods A. S. Localization and imaging of sialylated glycosphingolipids in brain tissue sections by MALDI mass spectrometry. Glycobiology 20, 661–667 (2010). 20190299 10.1093/glycob/cwq031 PMC2900884

[b16] Ikeda K., Shimizu T. & Taguchi R. Targeted analysis of ganglioside and sulfatide molecular species by LC/ESI-MS/MS with theoretically expanded multiple reaction monitoring. J. Lipid Res. 49, 2678–2689 (2008). 18703820 10.1194/jlr.D800038-JLR200

[b17] Li L. *et al.* Mass spectrometry methodology in lipid analysi*s*. Int. J. Mol. Sci. 15, 10492–10507 (2014). 24921707 10.3390/ijms150610492 PMC4100164

[b18] Takats Z., Wiseman J. M., Gologan B. & Cooks R. G. Mass spectrometry sampling under ambient conditions with desorption electrospray ionization. Science 306, 471–473 (2004). 15486296 10.1126/science.1104404

[b19] Wei Y. B. *et al.* Polystyrene spheres-assisted matrix-assisted laser desorption ionization mass spectrometry for quantitative analysis of plasma lysophosphatidylcholines. Anal. Chem. 85, 4729–4734 (2013). 23574540 10.1021/ac400452k

[b20] Lin L. *et al.* Ultra-high-performance liquid chromatography electrosprayionization tandem mass spectrometry for accurate analysis of glycerophospholipids and sphingolipids in drug resistance tumor cells. J. Chromatogr. A 1381, 140–148 (2015). 25614189 10.1016/j.chroma.2015.01.013

[b21] Wei Y. B. *et al.* A uniform 2,5-dihydroxybenzoic acid layer as a matrix for MALDI-FTICR MS-based lipidomics. Analyst 140, 1298–1305 (2015). 25568898 10.1039/c4an01964d

[b22] Fong B., Norris C. & McJarrow P. Liquid chromatography-high-resolution electrostatic ion-trap mass spectrometric analysis of GD(3) ganglioside in dairy products. Int. Dairy J. 21, 42–47 (2011).

[b23] Wallace W. E., Arnould M. A. & Knochemuss R. 2,5-Dihydroxybenzoic acid: laser desorption/ionisation as a function of elevated temperature. Int. J. Mass Spectrom. 242, 13–22 (2005).

[b24] Schiller J. *et al.* The suitability of different DHB isomers as matrices for the MALDI-TOF MS analysis of phospholipids: which isomer for what purpose? Eur. Biophys. J. 36, 517–527 (2007). 17047951 10.1007/s00249-006-0090-6

[b25] Metzger J. O., Woisch R., Tuszynski W. & Angermann R. New type of matrix for matrix-assisted laser desorption mass spectrometry of polysaccharides and proteins. Fresenius J. Anal. Chem. 349, 473–474 (1994).

[b26] Stahl B. *et al.* Oligosaccharides from human milk as revealed by matrix-assisted laser desorption/ionization mass spectrometry. Anal. Biochem. 223, 218–226(1994). 7887467 10.1006/abio.1994.1577

[b27] Fuchs B. *et al.* Phosphatidylcholines and -ethanolamines can be easily mistaken in phospholipid mixtures: a negative ion MALDI-TOF MS study with 9-aminoacridine as matrix and egg yolk as selected example. Anal. Bioanal. Chem. 395, 2479–2487 (2009). 19690837 10.1007/s00216-009-3032-1

[b28] Nonami H., Tanaka K., Fukuyama Y. & Erra-Balsells R. β-Carboline alkaloids as matrices for UV-matrix-assisted laser desorption/ionization time-of-flight mass spectrometry in positive and negative ion modes. Analysis of proteins of high molecular mass, and of cyclic and acyclic oligosaccharides. Rapid Commun. Mass Spectrom. 12, 285–296 (1998). 9534250 10.1002/(SICI)1097-0231(19980331)12:6<285::AID-RCM158>3.0.CO;2-4

[b29] Nonami H. *et al.* Evaluation of pyridoindoles, pyridylindoles and pyridylpyridoindoles as matrices for ultraviolet matrix-assisted laser desorption/ionization time-of-flight mass spectrometry. Rapid Commun. Mass Spectrom. 15, 2354–2373 (2001). 11746903 10.1002/rcm.514

[b30] Wang H. Y. J., Liu C. B. & Wu H. W. A simple desalting method for direct MALDI mass spectrometry profiling of tissue lipids. J. Lipid Res. 52, 840–849 (2011). 21266365 10.1194/jlr.D013060 PMC3284173

[b31] Floyd R. A. Antioxidants, oxidative stress, and degenerative neurological disorders. Proc. Soc. Exp. Biol. Med. 222, 236–245 (1999). 10601882 10.1046/j.1525-1373.1999.d01-140.x

[b32] Avdulov N. A. *et al.* Amyloid beta-peptides increase annular and bulk fluidity and induce lipid peroxidation in brain synaptic plasma membranes. J. Neurochem. 68, 2086–2091 (1997). 9109536 10.1046/j.1471-4159.1997.68052086.x

[b33] Murray I. V., Sindoni M. E. & Axelsen P. H. Promotion of Oxidative Lipid Membrane Damage by Amyloid β Proteins. Biochemistry 44, 12606–12613 (2005). 16156673 10.1021/bi050926p PMC2288524

[b34] Sayre L. M. *et al.* 4-Hydroxynonenal-derived advanced lipid peroxidation end products are increased in Alzheimer’s disease. J. Neurochem. 68, 2092–2097 (1997). 9109537 10.1046/j.1471-4159.1997.68052092.x

[b35] Markesbery W. R., Kryscio R. J., Lovell M. A. & Morrow J. D. Lipid peroxidation is an early event in the brain in amnestic mild cognitive impairment. Ann. Neurol. 58, 730–735 (2005). 16240347 10.1002/ana.20629

[b36] Gu F., Zhu M., Shi J., Hu Y. & Zhao Z. Enhanced oxidative stress is an early event during development of Alzheimer-like pathologies in presenilin conditional knock-out mice. Neurosci. Lett. 440, 44–48 (2008). 18539391 10.1016/j.neulet.2008.05.050

[b37] Lovell M. A., Ehmann W. D., Butler S. M. & Markesbery W. R. Elevated thiobarbituric acid-reactive substances and antioxidant enzyme activity in the brain in Alzheimer’s disease. Neurology 45, 1594–1601 (1995). 7644059 10.1212/wnl.45.8.1594

[b38] Vukelic Z., Metelmann W., Muthing J., Kos M. & Peter-Katalinic J. Anencephaly: structural characterization of gangliosides in defined brain regions. Biol. Chem. 382, 259–274 (2001). 11308024 10.1515/BC.2001.033

[b39] Metelmann W., Muthing J. & Peter-Katalinic J. Nano-electrospray ionization quadrupole time-of-flight tandem mass spectrometric analysis of a ganglioside mixture from human granulocytes. Rapid Commun. Mass Spectrom. 14, 543–550 (2000). 10775087 10.1002/(SICI)1097-0231(20000415)14:7<543::AID-RCM908>3.0.CO;2-J

[b40] Wishart D. S. *et al.* HMDB: the human metabolome database. Nucleic Acids Res. 35, D521–D526 (2007). 17202168 10.1093/nar/gkl923 PMC1899095

[b41] Wishart D. S. *et al.* HMDB: a knowledgebase for the human metabolome. Nucleic Acids Res. 37, D603–D610 (2009). 18953024 10.1093/nar/gkn810 PMC2686599

[b42] Wishart D. S. *et al.* HMDB 3.0—The human metabolome database in 2013. Nucleic Acids Res. 41, D801–D807 (2013). 23161693 10.1093/nar/gks1065 PMC3531200

