# Supplementary material for: Combination of ESI and MALDI mass spectrometry for qualitative, semi-quantitative and in situ analysis of gangliosides in brain
Source: Sci Rep. 2016 May 4;6:25289. doi: 10.1038/srep25289 (PMC4855142; doi:10.1038/srep25289)
Supplement: Supplementary Information [file srep25289-s1.doc]

**Supplementary Information for:**

**Combination of ESI and MALDI mass spectrometry for qualitative, semi-quantitative and in situ analysis of gangliosides in brain**

Yangyang Zhang, ^†^ Jun Wang, ^‡^ Jian’an Liu, ^†^ Juanjuan Han, ^†^ Shaoxiang Xiong, ^†^ Weidong Yong, ^‡^ Zhenwen Zhao^*,†,‖^

^†^ Beijing National Laboratory for Molecular Sciences, Key Laboratory of Analytical Chemistry for Living Biosystems, Institute of Chemistry Chinese Academy of Sciences, Beijing Mass Spectrum Center, Beijing, China

^‡^ Institute of Laboratory Animal Science, Chinese Academy of Medical Sciences & Peking Union Medical College, Beijing, China

^‖^ Graduate School, University of Chinese Academy of Sciences, Beijing, China

^*^**Corresponding author**: Phone: +86-10-62561239. Fax: +86-10-62561285. Email: [zhenwenzhao@iccas.ac.cn](mailto:zhenwenzhao@iccas.ac.cn).

Supplementary Figure S1. MS/MS spectrum of ganglioside d18:1/20:0 GM1.

Supplementary Figure S2. MS/MS spectrum of ganglioside d18:1/20:0 GD1.

Supplementary Figure S3. MS/MS spectrum of ganglioside d18:1/20:0 GT1.

Supplementary Figure S4. MS/MS spectrum of ganglioside d18:1/20:0 GQ1.

Supplementary Figure S5. MS/MS spectrum of ganglioside *O*-Acetyl d18:1/18:0 GD1.

Supplementary Figure S6. MS/MS spectrum of ganglioside *O*-Acetyl d18:1/18:0 GT1.

Supplementary Figure S7. MS/MS spectrum of ganglioside d18:1/18:0 GM2.

Supplementary Figure S8. MS/MS spectrum of ganglioside d18:1/18:0 GD3.

Supplementary Figure S9. MALDI MS/MS spectra of ganglioside d18:1/20:0 GM1 (A) and d18:1/18:0 GM1 (B).

Supplementary Figure S10: The mass spectrum of dried residue of ethanol after washing brain section obtained by MALDI FT-ICR MS in negative ion detection mode by using 3-AQ as matrix.


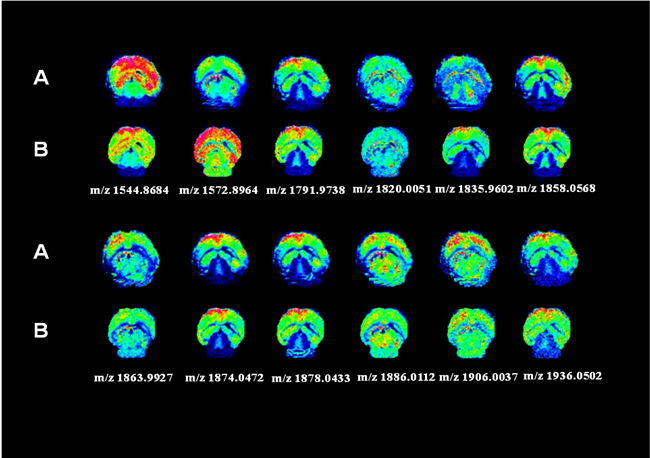


Supplementary Figure S11 The distribution of gangliosides with (B) or without (A) EtOH cleanup. Sectioned brain slices from control group were used.

Supplementary Table S1. Profiles of mouse brain gangliosides using UHPLC-ESI-FTICR MS.

| **Ganglioside** | ***m/z* Detected** | ***m/z* Exact** | **Mass accuracy /ppm** | **deprotonated molecule ion** | **Retention time/min** |
| --- | --- | --- | --- | --- | --- |
| d18:1/18:0 GM1 | 1544.8643 | 1544.8694 | -3.301 | [M-H]^-^ | 6.29 |
| d18:1/20:0 GM1 | 1572.8964 | 1572.9007 | -2.734 | [M-H]^-^ | 7.29 |
| d18:1/18:0 GM2 | 1382.8126 | 1382.8166 | -2.893 | [M-H]^-^ | 6.42 |
| d18:1/18:0 GM3 | 1179.7337 | 1179.7372 | -2.967 | [M-H]^-^ | 6.5 |
| d18:1/18:0 GD1 | 917.4773 | 917.4788 | -1.635 | [M-2H]^2-^ | 5.62 |
| d18:1/20:0 GD1 | 931.4925 | 931.4944 | -2.04 | [M-2H]^2-^ | 6.56 |
| *O*-Acetyl d18:1/18:0 GD1 | 938.4833 | 938.4841 | -0.852 | [M-2H]^2-^ | 5.56 |
| d18:1/18:0 GD3 | 1470.8261 | 1470.8326 | -4.419 | [M-H]^-^ | 6.22 |
| d18:1/18:0 GT1 | 1063.0242 | 1063.0265 | -2.164 | [M-2H]^2-^ | 5.09 |
| d18:1/20:0 GT1 | 1077.0397 | 1077.0421 | -2.228 | [M-2H]^2-^ | 6.09 |
| d18:1/22:0 GT1 | 1091.0542 | 1091.0578 | -3.3 | [M-2H]^2-^ | 7.09 |
| *O*-Acetyl d18:1/18:0 GT1 | 1084.0295 | 1084.0318 | -2.122 | [M-2H]^2-^ | 5.03 |
| *O*-Acetyl d18:1/20:0 GT1 | 1098.0441 | 1098.0474 | -3.005 | [M-2H]^2-^ | 6.03 |
| d18:1/18:0 GQ1 | 1208.5719 | 1208.5742 | -1.903 | [M-2H]^2-^ | 4.96 |
| d18:1/20:0 GQ1 | 1222.5862 | 1222.5898 | -2.945 | [M-2H]^2-^ | 5.95 |
